# 1856. Barriers to Improving Outcomes among People Experiencing Homelessness and People Who Inject Drugs Hospitalized for Complicated Infections

**DOI:** 10.1093/ofid/ofad500.1684

**Published:** 2023-11-27

**Authors:** Dana M Parke, Rachel M Kenney, John Bogojevich, Caren El-Khoury, Seema Joshi, Simran Brar, Lynsey MacDonald, Christina Salib, Nancy MacDonald, Michael Veve, Geehan Suleyman

**Affiliations:** Henry Ford Health, Detroit, Michigan; Henry Ford Hospital, Detroit, Michigan; Henry Ford Health, Detroit, Michigan; Henry Ford Health, Detroit, Michigan; Henry Ford Hospital, Detroit, Michigan; Wayne State School of Medicine, Detroit, Michigan; Henry Ford Hospital, Detroit, Michigan; Henry Ford Health, Detroit, Michigan; Henry Ford Hospital, Detroit, Michigan; Henry Ford Health, Detroit, Michigan; Henry Ford Health, Detroit, Michigan

## Abstract

**Background:**

People experiencing homelessness (PEH) and people who inject drugs (PWID) experience health disparities and worse outcomes. Challenges include suboptimal medication use, loss to follow-up, and non-compliance due to social determinant of health (SDOH) barriers, including lack of stable housing and transportation, limited financial resources, substance use, and addiction.

**Methods:**

This quality improvement project aimed to address SDOH barriers among hospitalized PEH and/or PWID requiring ≥ 2 weeks of antibiotics to improve antibiotic compliance and outcomes in Detroit from 6/2022-4/2023. Interventions included antibiotic education, addiction medicine and pharmacy discharge medication cost inquiry consults when indicated, ensuring oral antibiotics were in hand at discharge, strengthening discharge planning between inpatient and ambulatory case managers (ACM), and referrals to community-based organizations to address SDOH needs.

**Results:**

34 patients were included (8 PEH, 11 PWID, 15 both); 3 who died in the hospital were excluded. Multiple individual and structural barriers and challenges to improving adherence and outcomes were identified (Table 1). Loss to follow-up was a significant challenge among this cohort, primarily due to patients self-discharging (29%) and being unreachable (52%). 10 (37%) patients were offered SDOH services (Table 2). Patients also had significant behavioral health/substance use disorder needs and utilized healthcare at a very high rate, with 29% having an ED revisit and 44% being readmitted within 30 days after discharge. Several structural and SDOH barriers existed, including limited staff capacity and limited placement options after discharge, resulting in suboptimal treatment delivery.

**Figure:**
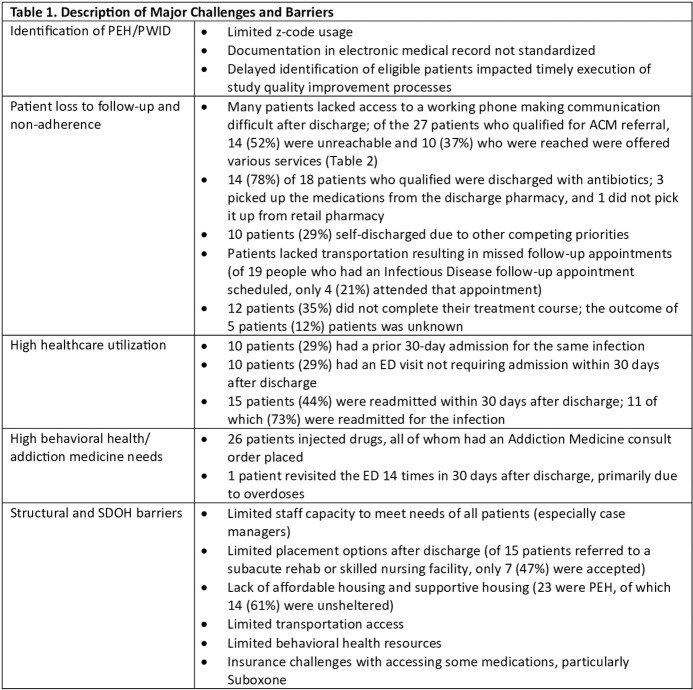

**Figure:**
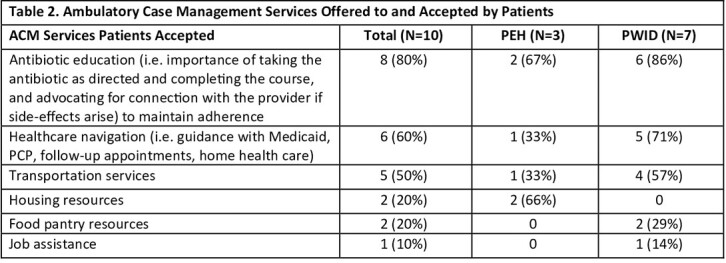

**Conclusion:**

Addressing SDOH barriers for PEH and PWID is challenging but vital to improving outcomes. Qualitative research should be conducted to understand these barriers. Having an interdisciplinary team comprising of infectious diseases, pharmacy, addiction medicine, case management and population health is critical to address patient needs holistically. Strengthening internal processes and building additional community-based partnerships will be essential to better meet patient needs after discharge.

**Disclosures:**

**Michael Veve, PharmD, MPH**, National Institutes of Health: Grant/Research Support|Paratek Pharmaceuticals: Grant/Research Support

